# Investigating the consequences of asymmetric endoplasmic reticulum inheritance in *Saccharomyces cerevisiae* under stress using a combination of single cell measurements and mathematical modelling

**DOI:** 10.1016/j.synbio.2018.01.001

**Published:** 2018-01-17

**Authors:** Felix R.H. Jonas, Kate E. Royle, Rochelle Aw, Guy-Bart V. Stan, Karen M. Polizzi

**Affiliations:** a Department of Bioengineering, Imperial College London, London, SW7 2AZ, United Kingdom; b Imperial College Centre for Synthetic Biology, London, SW7 2AZ, United Kingdom; c Department of Life Sciences, Imperial College London, London, SW7 2AZ, United Kingdom

**Keywords:** Adaptation, Asymmetric cell division, Endoplasmic reticulum stress, Unfolded protein response, *Saccharomyces cerevisiae*

## Abstract

Adaptation allows organisms to maintain a constant internal environment, which is optimised for growth. The unfolded protein response (UPR) is an example of a feedback loop that maintains endoplasmic reticulum (ER) homeostasis, and is characteristic of how adaptation is often mediated by transcriptional networks. The more recent discovery of asymmetric division in maintaining ER homeostasis, however, is an example of how alternative non-transcriptional pathways can exist, but are overlooked by gold standard transcriptomic or proteomic population-based assays. In this study, we have used a combination of fluorescent reporters, flow cytometry and mathematical modelling to explore the relative roles of asymmetric cell division and the UPR in maintaining ER homeostasis. Under low ER stress, asymmetric division leaves daughter cells with an ER deficiency, necessitating activation of the UPR and prolonged cell cycle during which they can recover ER functionality before growth. Mathematical analysis of and simulation results from our mathematical model reinforce the experimental observations that low ER stress primarily impacts the growth rate of the daughter cells. These results demonstrate the interplay between homeostatic pathways and the importance of exploring sub-population dynamics to understand population adaptation to quantitatively different stresses.

## Graphical abstract

**Figure:**
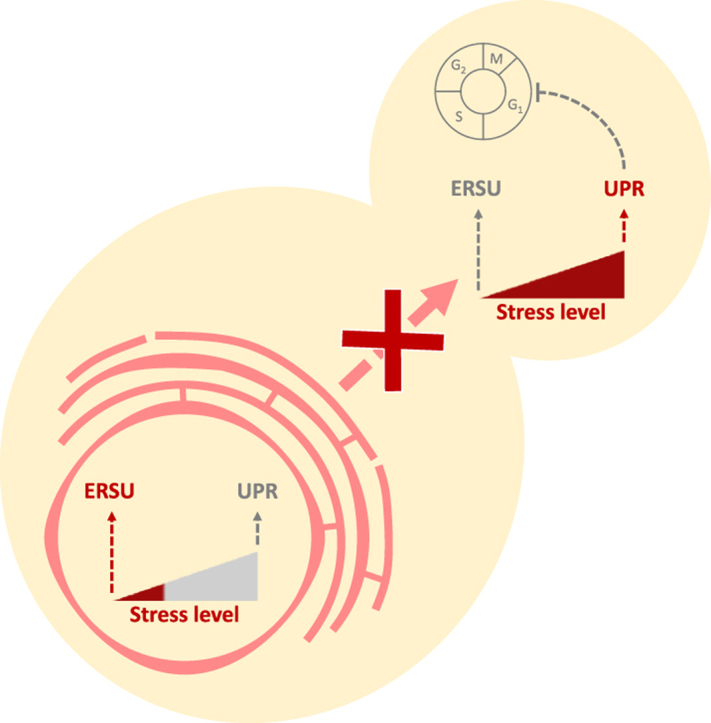

## Abbreviations

ACT1: Actin 1
ConA: TRITC-labelled concanavalin A
DTT: Dithiothreitol
ER: Endoplasmic reticulum
ERAD: ER associated degradation
ERSU: ER stress surveillance pathway
ERO1: Endoplasmic Reticulum Oxidoreductin 1
eroGFP: ER-targeted redox-sensitive GFP
GFP: Green fluorescent protein
OD_600_: optical density at 600 nm
ODE: Ordinary Differential Equation
Tm: Tunicamycin
UPR: Unfolded protein response
YPD: Yeast Peptone Dextrose medium

## 1 Introduction

Adaptation is the basic mechanism that enables organisms to thrive in a changing, and often challenging, environment. Both single and multicellular organisms have evolved a set of internal conditions that allow them to fully exploit an ecological niche. Organisms maintain this homeostasis by adapting: different stresses are detected by specific sensors, which trigger bespoke transcriptional responses [1]. This regulation of gene networks collectively acts to reinstate homeostasis, be it through an adjustment of metabolism [2], cellular transport [3], or motility [4]. One example of adaptation is the maintenance of endoplasmic reticulum (ER) homeostasis. The ER, a large organelle comprising a single lipid bilayer and enclosed lumen, extends as a network throughout cell and is responsible for a diverse range of functionalities including: (i) protein synthesis, folding and quality control, (ii) calcium storage and (iii) lipid metabolism [5], [6], [7].

**Fig. 1:**
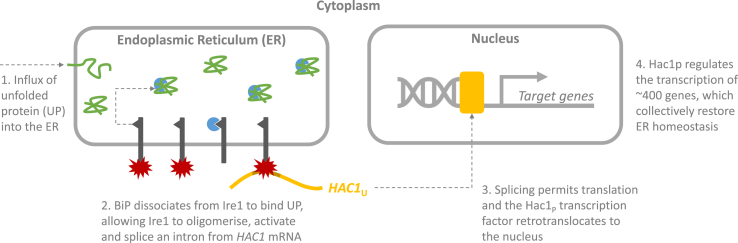
**Unfolded protein response (UPR) signalling.** The UPR is a transcriptional response to deviations in endoplasmic reticulum (ER) homeostasis. For instance, an influx of unfolded protein (green) to the ER causes the chaperone BiP (blue) to dissociate from the transmembrane stress sensor Ire1 (dark grey) to help protein folding. Ire1 subsequently oligomerises and activates (red), allowing its cytoplasmic RNAse domain to cleave an intron (brown) from the *HAC1* mRNA (yellow) permitting translation. The Hac1p transcription factor retrotranslocates to the nucleus where it regulates the transcription of around 400 genes, which act collectively to restore ER homeostasis.

Focusing on protein synthesis, when nascent polypeptides enter the ER they interact with a range of chaperones and foldases that direct protein folding, and ensure only those with the native conformation progress through the secretory pathway. During high protein expression or in sub-optimal environments, however, these pathways are overloaded and the ER becomes crowded with unfolded and misfolded proteins, activating the unfolded protein response (UPR, Fig. 1). The stress sensor in this case is the transmembrane protein Ire1, and in combination with the chaperone BiP (Kar2 in yeast) and the transcription factor Hac1p, constitute the highly conserved components of the eukaryotic unfolded protein response (UPR) [8], [9]. The excess unfolded protein sequesters BiP, causing dissociation of the lumenal Ire1-BiP complex. This allows Ire1 to oligomerise, activating its cytoplasmic RNAse domain, which in turn cleaves an intron from the *HAC1* mRNA permitting translation [10], [11]. The Hac1p transcription factor retrotranslocates to the nucleus where it regulates the transcription of around 400 genes, associated with protein trafficking and quality control, metabolism, and cell wall biosynthesis, which collectively restore ER homeostasis [12].

The importance of adaptation mechanisms to these deviations can be inferred through the prevalence of biological redundancy, conferring robustness. In this instance, the role of the UPR in maintaining ER homeostasis is so critical that additional branches have evolved in higher eukaryotes [13], and the network is often implicated in diseases such as neurodegeneration [14], viral infection [15], and cancer [16]. This redundancy does, however, complicate our understanding of the system and increases the importance of knowing not only the identity of the pathways, but in determining their relative roles and interactions [17]. Although research into the UPR has elucidated the molecular interactions of Ire1 and BiP [18], [19], [20], links to other regulatory pathways and the presence of additional mechanisms are to be expected. This is particularly pertinent in low and medium stress conditions: most environmental changes are not binary in nature but continuous, and therefore, cells may use a variety of different mechanisms including those that operate without the need to activate changes in gene expression.

One such adaptation mechanism which is becoming increasingly apparent is to trigger asymmetric division of organelles [21]. Research in this field has focused on understanding how these complex structures, including the mitochondria and vacuole, are divided between mother and daughter yeast cells as *de novo* generation is often slow – and in cases such as the ER, impossible [22]. There is now an increasing precedent for asymmetric division of ER under stress, particularly with the discovery that mother cells can retain a greater majority of damaged components during budding through the ER surveillance (ERSU) pathway [23]. This mechanism is independent of the UPR and operates through the MAP kinase Slt2, along with components of the cell wall integrity pathway, to delay the passage of damaged ER to daughter cells [24] through the formation of a lipid barrier at the bud neck [25]. This delay extends cytokinesis until a minimal threshold of ER functional capacity is reached, ensuring mother cell viability [23].

Here, we sought to understand the roles of asymmetric division and UPR activation in population adaptation to low ER stress. In research scenarios, ER stress is frequently induced with high (mM) concentrations of chemical inhibitors, such as DTT or tunicamycin, to ensure strong activation in all cells [26]. This has been vital for understanding the molecular basis of these pathways, but reveals the mechanisms under extreme conditions. Here, we decreased the concentration of tunicamycin from the typical (2 μg/mL) to a more physiologically relevant value (100 ng/mL) based on the IC_50_ value of its target, *ALG7* [27], [28]. Furthermore, in place of more traditional population-wide readouts such as RNA splicing, qRT-PCR, and Western blotting for phosphorylation changes [26], we used a combination of fluorescent reporters and flow cytometry to obtain single cell data. We coupled this data to mathematical modelling to subsequently explore the consequences of asymmetric ER inheritance for population growth rate changes, and therefore, adaptation.

## 2 Material and methods

### 2.1 Yeast strains and plasmids

All yeast strains and plasmids used in this study are summarized in Appendix A. The reporter plasmids for UPR activation and ER content were derived from pRS403 [29], pPM28 (Addgene #20131), pPM47 (Addgene #20132) [30] and pMaM175 [31] via standard molecular biology techniques (overlap extension PCR and restriction enzyme based cloning) and verified by DNA sequencing (Source Biosciences, Nottingham, UK). Yeast were transformed using the lithium acetate method [32], followed by plating on histidine dropout selective medium. Colonies were verified by colony PCR.

### 2.2 Cell growth, ER stress induction, synchronisation, and Concanavalin A staining

Overnight cultures were diluted to an OD_600_ of 0.1 in prewarmed YPD medium. After 6–8 h of growth at 30 °C, cells were either immediately exposed to tunicamycin at the stated concentration or first synchronised with nocadozole and/or stained with TRITC-labelled Concanavalin A (ConA) before exposure.

For population synchronisation, nocadozole, dissolved in DMSO, was added to exponentially growing cells to a final concentration of 15 μg/mL and incubated for 2 h before staining.

For ConA staining, 0.3–0.5 mL of exponentially growing cells were collected by centrifugation at 2600 rpm in a benchtop microcentrifuge for 90 s, washed once in PBS and then incubated for 15 min at room temperature in staining solution (0.1 mg/mL TRITC-ConA in PBS). Afterwards, cells were washed once and resuspended in 0.5 mL prewarmed YPD medium before diluting them 1:10 to prewarmed medium containing tunicamycin.

### 2.3 Flow cytometry assay

Growing cells were collected at the indicated time points and resuspended 1:7–1:10 in cold ddH_2_0 (for UPR reporter measurements) or 11 mM DTT in ddH_2_O (for eroGFP content measurements in order to obtain fully reduced eroGFP with maximal, environment-independent fluorescence). Optical properties were collected using a BD FACScan™ flow cytometer (BD Biosciences, Oxford, UK) and an attached Automatic Multisampler from Cytek™ (Fremont, CA, USA). Data was collected using Cellquest V™ (BD Biosciences, Oxford, UK). GFP was excited with a 488 nm laser and emission was measured at 510 nm (FL1-H). Red fluorescence (TRITC-ConA) was excited at 561 nm and emission was measured at 600 nm (FL-4 H). Data was analysed with the FlowJo™ software (Ashland, Oregon, USA) and reported measurements are the result of at least 3 independent biological replicates (n ≥ 3).

### 2.4 Reverse-transcription quantitative PCR (qRT-PCR)

RNA for qRT-PCR was isolated using RiboPure-Yeast Kit, according to manufacturer's instructions (Applied Biosystems, Warrington, UK), from ∼10^7^ cells. The RNA concentration was determined via spectroscopy with a Nanodrop 1000 (ThermoScientific, Hemel Hempstead, UK) and 1 μg was converted to cDNA using the Tetro™ cDNA Synthesis Kit (BioLine, London, UK) according to the manufacturer's instructions. qRT-PCR reactions were set up using the 2× SYBR^®^ Green JumpStart Taq Ready Mix (Sigma-Aldrich, Dorset, UK) and amplified in a Mastercycler *realplex* (Eppendorf UK Ltd, Histon, UK) using the following thermocycling conditions: 95 °C for 5 min, 40 cycles of 95 °C for 15 s and 63.4 °C for 40 s, and a melting curve of 98 °C–25 °C for 15 min. Data was analysed using the ΔΔ−Ct method [33] and normalised to *ACT1* as the housekeeping gene. Primer pairs were: *ACT1* – CATGAAGTGTGATGTCGATGTCCGT and CGGCAATACCTGGGAACATGGTGG; *HAC1* total – TGCGACGATATAGCGGGAAACAGT and TTCCTGGTCATCGTAATCACGGCT and *ERO1* – TCCGGTTTCCATGCCTCTATCGGT and TCCAGATTGGGCTCCCATTTACCA.

### 2.5 Fluorescence microscopy and budding index determination

Four hours after TRITC-ConA staining and tunicamycin exposure, 0.5 mL of cells were collected, resuspended in PBS and imaged on a Ti Eclipse microscope (Nikon, Alton, UK) with TRITC filter set. NIS - Elements™ (Nikon, Alton, UK) was used for visualisation and budding index was determined after counting at least 100 cells for each condition and biological replicate (n = 3).

### 2.6 Long-term population growth in microtitre plates

A method for monitoring long-term growth rates was adapted from Toussaint and Cocconi [34]. Exponentially growing cells (OD∼0.5) were diluted 1:50 into 100 μl of YPD medium in 96-well clear flat-bottomed plates (Costar, Corning Inc, UK) with or without the addition of tunicamycin. Continuous OD_600_ measurements over 8 h of incubation were conducted using a Synergy HT Platereader (Biotek Instruments, Potton, UK). Growth parameters (relative population growth rate and tunicamycin induced lag time) of three technical replicates were determined by fitting.

## 3 Theory

### 3.1 Mother and daughter populations can be represented by a linear, time-invariant ordinary differential equation (ODE) model of order two

The cytokinesis of mother *or* daughter cells results in one mother and one daughter cell. Thus, the proliferation rate of one subpopulation can be represented by the cell division rate of the other subpopulation. This results in the following linear, time-invariant, second order ODE model:

(1) $$M^{\prime }\left(t\right)=\frac{dM\left(t\right)}{dt}=k_{D}D\left(t\right)-d_{M}M\left(t\right)$$

(2) $$D^{\prime }\left(t\right)=\frac{dD\left(t\right)}{dt}=k_{M}M\left(t\right)-d_{D}D\left(t\right)$$

where, $k_{D}$ is the cell division rate of the daughters, $k_{M}$ is the division rate of the mothers, $d_{D}$ is the death rate of the daughters, and $d_{M}$ is the death rate of the mothers.

The solution of such a second order linear time-invariant ordinary differential equation model can be obtained straightforwardly [35].

**Fig. 2:**
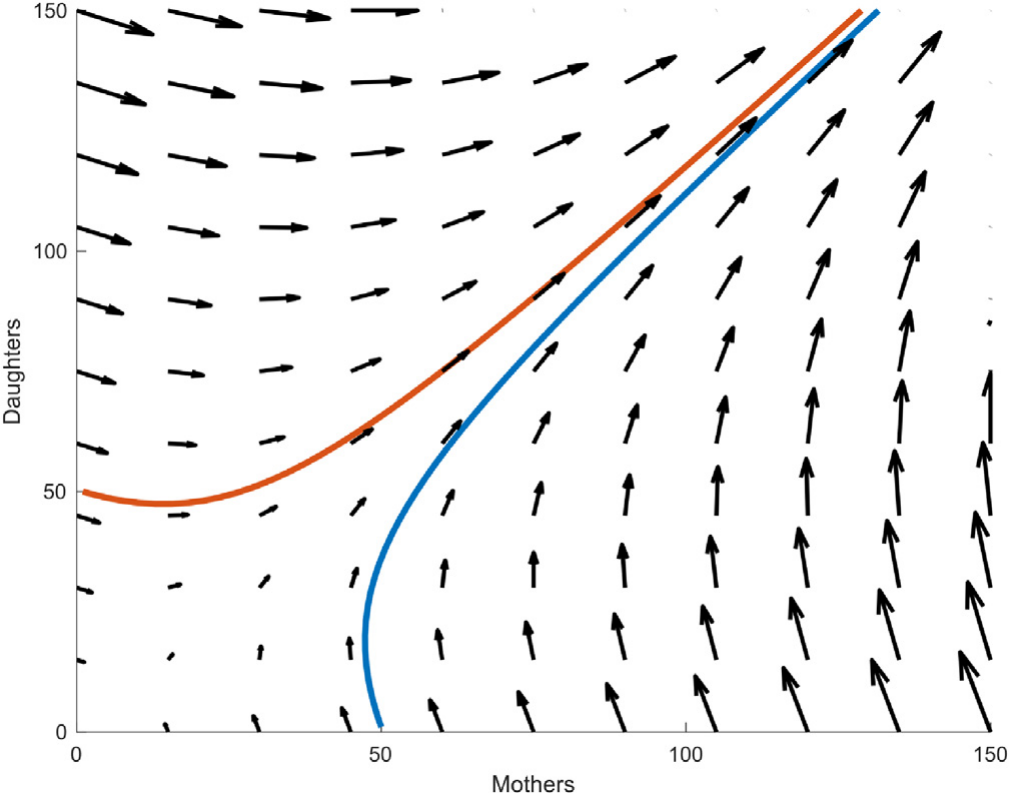
**Phase portrait of the mother-daughter population dynamics after asymmetric cell division.** Trajectory of the mother and daughter subpopulations in the mother-daughter phase plane for two different initial conditions. The small arrows represent the vector field associated with the mother-daughter population dynamics described in equations (1), (2). The simulations were executed in Matlab using two sets of initial conditions (50 mothers, 1 daughter and 50 daughters, 1 mother) with ${\text{k}}_{\text{M}}$ = 2, ${\text{k}}_{\text{D}}$ = 1.5.${\text{d}}_{\text{M}}={\text{d}}_{\text{D}}=0.6$.

An example of a numerical simulation of system (1)–(2) starting from the initial conditions (M(0),D(0))=(1,50) and (M(0),D(0))=(50,1) under the condition ${\text{k}}_{\text{M}}{\text{k}}_{\text{D}}\geq {\text{d}}_{\text{M}}{\text{d}}_{\text{D}}$ (${\text{k}}_{\text{M}}$=2, ${\text{k}}_{\text{D}}$ = 1.5, ${\text{d}}_{\text{M}}={\text{d}}_{\text{D}}$ = 0.6) is given in Fig. 2.

As can be seen from this illustrative simulation, although both M(t) and D(t) increase over time, their ratio seems to converge to a defined value. In what follows, we more precisely characterise this convergence property of the daughter-to-mother ratio.

### 3.2 The daughter-to-mother ratio (r) converges asymptotically to a constant value given by the geometric mean of the growth rates of the subpopulations minus their death rate

First, we define the ratio of daughter-to-mother cells as

$$r\left(t\right)=\frac{D\left(t\right)}{M\left(t\right)}$$

The dynamics of the daughter-to-mother ratio r(t) is given as:

$$r^{\prime }\left(t\right)=\frac{dr\left(t\right)}{dt}=\frac{MD^{\prime }-DM^{\prime }}{M^{2}}=-k_{D}r^{2}+\left(d_{M}-d_{D}\right)r+k_{M}$$

where the second equality is easily obtained using Equations (1) and (2) above.

At steady-state, $r^{\prime }\left(t\right)=0$, which leads to:

$$k_{D}r_{ss}^{2}+\left(d_{D}-d_{M}\right)r_{ss}-k_{M}=0$$

where $r_{ss}$ is the daughter-to-mother ratio at steady-state.

The only positive solution to this quadratic equation is:

(3) $$r_{ss}\left(d_{M},d_{D},k_{D},k_{M}\right)={\left(D/M\right)}_{ss}=\frac{\left(d_{D}-d_{M}\right)+\sqrt{{\left(d_{D}-d_{M}\right)}^{2}+4k_{D}k_{M}}}{2k_{D}}$$

As long as $k_{M}k_{D}\geq d_{M}d_{D}$, that is, as long as cells are dividing faster than they are dying, both mother and daughter populations will monotonically increase with time and the daughter-to-mother ratio will asymptotically converge to the value $r_{ss}$ in (3).

The total population growth rate per cell, $k_{P}$, can be expressed in terms of the daughter-to-mother ratio r as follows:

$$k_{P}=\frac{D^{\prime }\left(t\right)+M^{\prime }\left(t\right)}{D\left(t\right)+M\left(t\right)}=\frac{k_{M}M+k_{D}D-d_{M}M-d_{D}D}{M+D}=\frac{\left(k_{M}+k_{D}r\right)-\left(d_{M}+d_{D}r\right)}{1+r}$$

If we assume that the daughter-to-mother ratio has reached its stable steady-state value, i.e. if we assume that $r=r_{ss}$, the total population growth rate per cell, i.e. $k_{P}=\frac{D^{\prime }\left(t\right)+M^{\prime }\left(t\right)}{D\left(t\right)+M\left(t\right)}$ is then given by:

(4) $$k_{P}\left(r=r_{ss}\right)=\frac{\left(k_{M}+r_{ss}k_{D}\right)-\left(d_{M}+r_{ss}d_{D}\right)}{1+r_{ss}}$$

If we assume similar death rates in both mother and daughter subpopulations, i.e. $d_{D}=d_{M}=d_{P}$ (A1), the steady-state population ratio simplifies to:

(A1) in (3):

(5) $$r_{ss}\left(d_{D}=d_{M}=d_{P}\right)=\frac{\left(d_{D}-d_{M}\right)+\sqrt{{\left(d_{D}-d_{M}\right)}^{2}+4k_{D}k_{M}}}{2k_{D}}=\sqrt{\frac{k_{M}}{k_{D}}}$$

which leads to a total population growth rate per cell given by:

(6) $$k_{P}\left(r=r_{ss},d_{D}=d_{M}=d_{P}\right)=\frac{k_{M}+r_{ss}k_{D}}{1+r_{ss}}-d_{P}=\frac{k_{M}+\sqrt{\frac{k_{M}}{k_{D}}}k_{D}}{1+\sqrt{\frac{k_{M}}{k_{D}}}}-d_{P}=\sqrt{k_{M}k_{D}}-d_{P}$$

This means that, at steady-state, under the assumptions above, i.e. $k_{M}k_{D}\geq d_{M}d_{D}$ and $d_{D}=d_{M}$, the growth rate per cell of the whole population is given by the geometric mean of the growth rates of the subpopulations minus their common death rate.

### 3.3 Calculating growth rates for the individual subpopulations from experimental data

Experimental measurements of cell growth rates (e.g. optical density, colony forming unit counts, etc) largely measure the population net growth rate, $k_{P}.\;$ Therefore, it is useful to be able to calculate the growth rates of the subpopulations from $k_{P}.\;$

Following on from Equation (6), if the cell death rate is negligible ($d_{P}=0$), then

$$k_{P}\left(r=r_{ss},d_{D}=d_{M}=d_{P}=0\right)=\sqrt{k_{M}k_{D}}$$

If we assume that the growth rates of mothers and daughters are proportional to each other, with an asymmetry factor a providing the proportionality constant, then we have:

$$k_{D}=ak_{M}$$

$$\text{As}\;\text{a}\;\text{consequence}:\;k_{P}\left(r=r_{ss},d_{D}=d_{M}=d_{P}=0\right)=\sqrt{ak_{M}^{2}}$$

Therefore, if we know the population growth rate $k_{P}$, we can deduce

(7) $$k_{M}=\frac{1}{\sqrt{a}}k_{p}$$

(8) $$k_{D}=ak_{M}=\sqrt{a}k_{p}$$

## 4 Results

### 4.1 UPR is not necessary for survival during low ER stress, but both UPR and ERSU contribute to adaptation

**Fig. 3:**
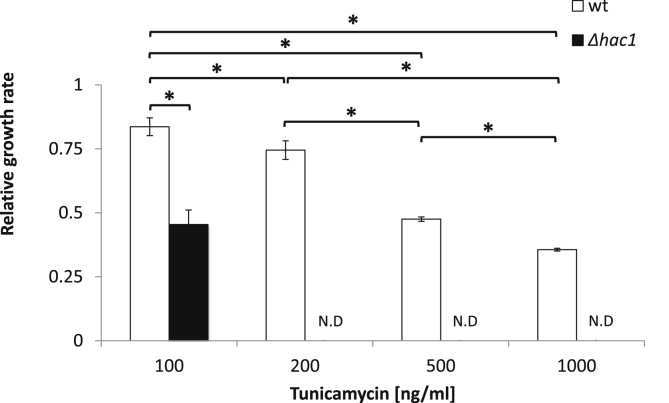
**Relative growth rate of wild-type and UPR-deficient (Δhac1) strains during treatment with increasing concentrations of tunicamycin.** All strains were cultivated in 100 μL YPD in 96-well plates and continuous OD_600_ measurements were taken over 8 h. Growth rate was calculated for each tunicamycin concentration relative to the growth rate in YPD medium supplemented with DMSO (the solvent control); errors bars indicate standard deviation. Student's t-tests were carried out to determine significant differences in growth rate between strains and conditions; asterisks indicate a p-value < .05 (n = 3). ‘N.D’: not detectable.

To understand the relative roles of asymmetric division and UPR activity in yeast population adaptation to ER stress, we initially sought to validate the interaction between the two. As the UPR is mediated by the Hac1p transcription factor, both wild-type and Δ*hac1* cells were treated with a range of tunicamycin concentrations (100, 200, 500 and 1000 ng/mL) and the relative growth rate was measured (Fig. 3). While wild-type yeast cells could grow at all concentrations of tunicamycin, growth of the Δ*hac1* cells was only detected at 100 ng/mL. This both highlights the importance of UPR activity in adaptation to ER stress, but reveals redundancy in ER homeostasis as non-UPR mechanisms also contribute to adaptation at low (TM100) levels of ER stress.

### 4.2 The ER is asymmetrically inherited under low ER stress conditions via activation of the ERSU pathway and the same lipid dependent diffusion barrier that operates under high stress conditions

Considering the activity of non-UPR adaptation mechanisms, we sought to validate asymmetric inheritance under low ER stress conditions. To this end, we constitutively expressed the ER-targeted redox-sensitive GFP (eroGFP) as a measure of the average content of the ER in yeast cells. The sensor comprises the redox-sensitive roGFP2 with a C-terminal ER retrieval sequence, HDEL, which localises the reporter to the ER [30]. The excitation maximum of eroGFP changes with the redox state of the disulphide bonds and therefore enables the measurement of the average redox state of the ER. We selected eroGFP as a reporter to enable us to both measure total ER content and examine whether there were changes in the redox potential between mother and daughter cells. However, preliminary experiments suggested that photobleaching was occurring during the redox measurements (which require excitation of the cells at two different wavelengths). Therefore, we endeavoured to use the reporter solely as a measure of total ER content. To ensure our measurements were a proxy of total size, rather than redox state, we treated the cells with a bolus addition of DTT to reduce the entire population of eroGFP molecules and minimise excitation wavelength changes across the population. To account for variations in eroGFP fluorescence due to stress, we included a DTT only control population in our measurements.

**Fig. 4:**
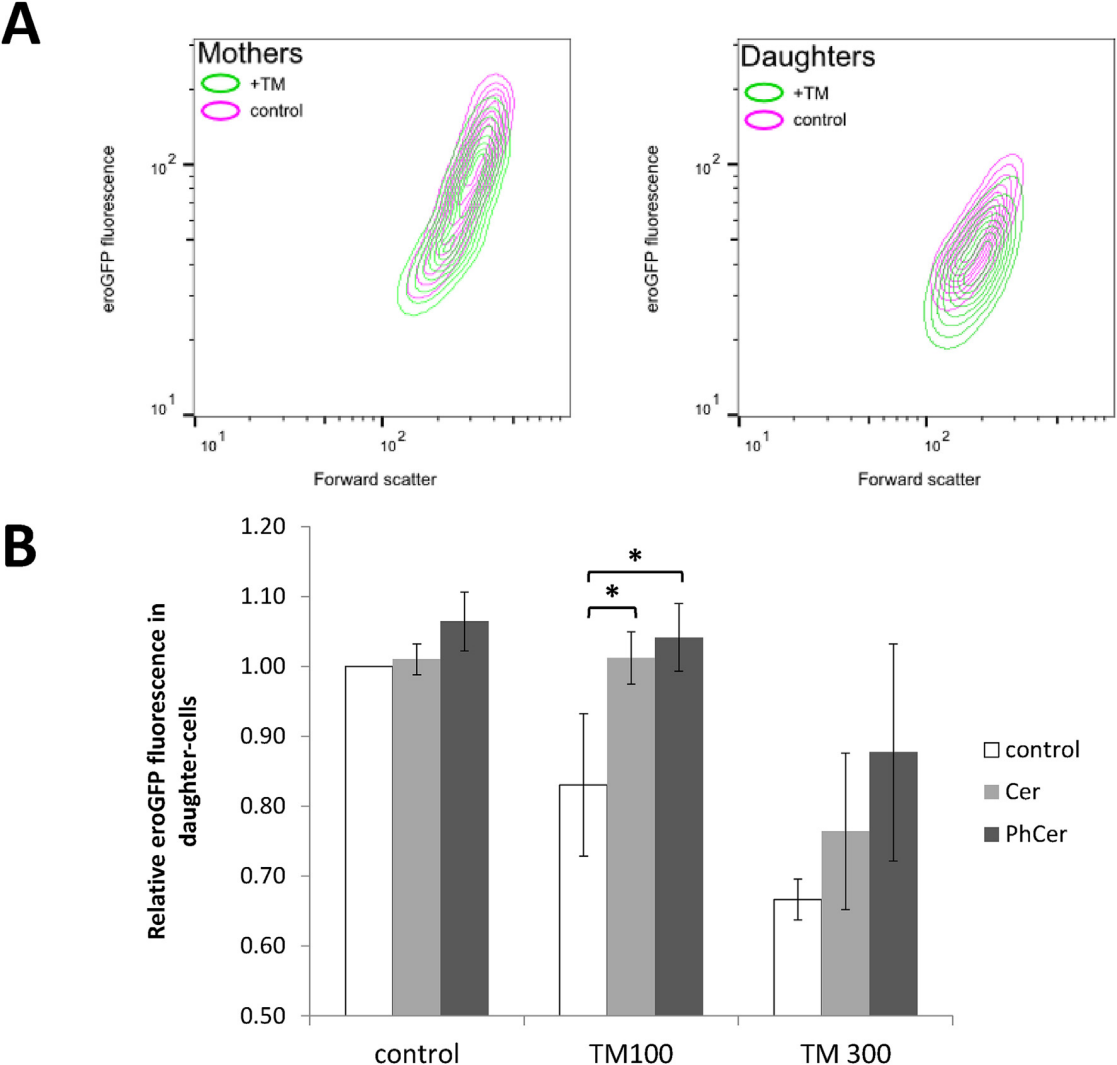
**Asymmetric division of ER between mother and daughter cells under low stress conditions.** [A] The effect of tunicamycin treatment (green) on eroGFP and cell size (as indicated by forward scatter) between mother and daughter cells, in comparison to the DMSO control (pink). [B] The effect of ceramide (Cer) and phytoceramide (PhCer) supplementation on eroGFP fluorescence in daughter cells during no (DMSO), low (TM100) and high (TM300) stress. Relative eroGFP values were calculated as the ratio between the median eroGFP fluorescence under each treatment in comparison to the median eroGFP fluorescence in YPD; errors bars indicate standard deviation (n = 3). Student's *t*-tests were carried out to determine significant differences in growth rate between strains and conditions; asterisks indicate a p-value < .05.

To separate the mother and daughter populations, yeast cells were synchronised at the end of G2 phase with nocodazole and stained with Concanavalin A (ConA). This lectin binds to the sugars of the yeast cell wall [36]; following cytokinesis, the cell wall remains with the mother, as such the stain can be used to differentiate pre-existing cells (mothers) from cells which were born later (daughters). Our hypothesis, therefore, was that asymmetric inheritance under low ER stress conditions would lead to differences in total eroGFP between mother and daughter cells. Flow cytometry confirmed this, revealing less eroGFP fluorescence in daughter cells when compared to mother cells, and a smaller proportional amount when cell size is accounted for (Fig. 4 [A]). This shows asymmetric division of the ER still occurs under low stress conditions, in agreement with the previous observation that mother cells harbour damaged ER components through the ERSU pathway during high stress [23]. Indeed, when the cells were supplemented with ceramide or phytoceramide to disrupt formation of the required lipid barrier for the ERSU pathway [25], the previously observed differences in ER content between mother and daughter cells were abolished (Fig. 4 [B]). Collectively, these results show that the same pathways operate under low ER stress conditions as in high.

### 4.3 After asymmetric division, ER stress is primarily activated in daughter cells under low stress conditions, which lengthens the daughter delay

**Fig. 5:**
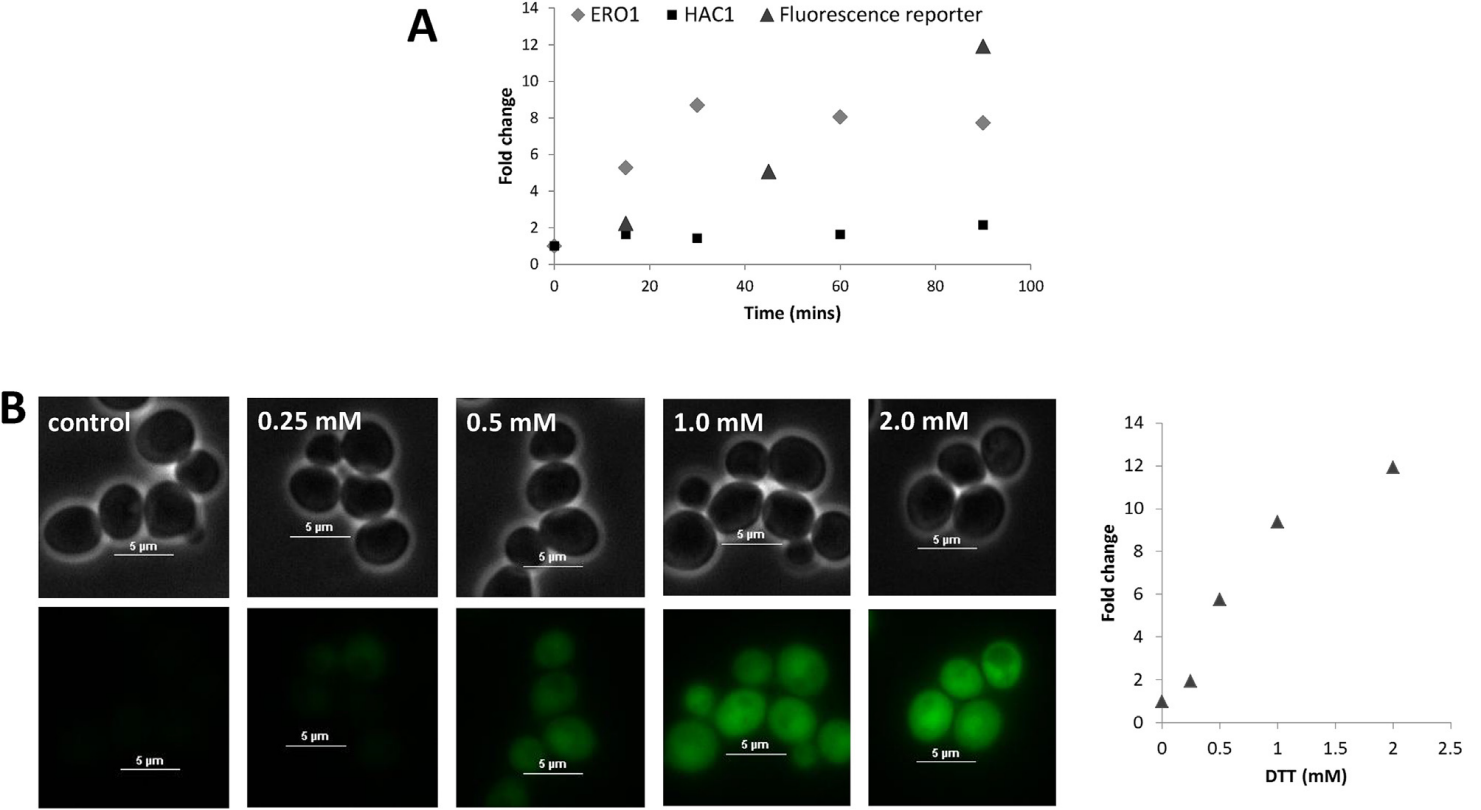
**Experimental validation of the *HAC1* fluorescent reporter**. [A] Comparison of mRNA (*ERO1*, *HAC1*) and a fluorescent reporter for UPR stress. Cells in the exponential phase were treated with 2 mM DTT, a potent chemical inducer of ER stress, and mRNA/fluorescence levels measured over 90 min. The fold change in mRNA targets was calculated relative to the fold change in an internal standard, *ACT1*. [B] Fluorescent reporter response to increasing concentrations of inducer. Cells in the exponential phase were treated with different concentrations of inducer and analysed by fluorescence microscopy (left) and flow cytometry (right) after 90 min of exposure. Linear regressions were carried out on the fold change in reporter fluorescence over time [A] and DTT concentration [B], with R^2^ values of 0.9857 and 0.9016 respectively.

To understand the consequences of asymmetric inheritance on ER homeostasis, we sought to measure the activation of the UPR at different concentrations of tunicamycin. Standard techniques for analysing UPR activation dynamics are both aggregate measurements and terminal assays, but here we are concerned with the role of ER stress in adaptation, and consequently needed a technique that would allow for single, live cell, measurements from which we could derive subpopulation dynamics during growth. To this end, we developed an improved single cell reporter of UPR with fast folding and degradation dynamics by fusing a ubiquitin domain to a yeast codon-optimised superfolder GFP [37], [38], [39], [40], [41] (Appendix A). This allowed more accurate monitoring of fluctuations when compared to previous versions of the reporter [30]. Having confirmed that the reporter did not substantially alter the exponential growth rate in YPD medium (μ_WT_ = 0.0081 min^−1^; μ_Δ*hac1*_ = 0.0078 min^−1^), we tested its agreement with qRT-PCR measurements during treatment with DTT, a potent chemical inducer of stress (Fig. 5 [A]). The reporter utilises the promoter for the *HAC1* gene, which is self-regulated by its own spliced transcription factor [42]. We therefore compared the fold-change in fluorescence of the reporter to that of the fold-change in mRNA levels of the *HAC1* gene. Initially, we observed a similar quantitative increase in both qRT-PCR and reporter measurements during early time points (up to 15 min). However, thereafter the qRT-PCR measurements seem to saturate, whereas the reporter fluorescence continued to increase. This saturation is suggestive of an upper threshold of gene regulation with prolonged exposure. To determine if this was gene specific, we also examined the change in expression levels of another target regulated by the Hac1p transcription factor, *ERO1*. Once again, the fold-change in mRNA levels saturated early in the time-course measurement suggesting a more general phenomenon. As changes in mRNA abundance are calculated with respect to changes in a housekeeping gene (here, *ACT1*), any increase in growth from 15 to 90 min would upregulate *ACT1* expression and concomitantly decrease calculated changes in target abundance. Consequently, we have developed an improved tool for quantifying UPR activity in single yeast cells which shows a larger linear dynamic region (R^2^ = 0.9857), enabling more accurate quantitative measurements. We also analysed the dynamic range of the reporter with respect to the concentration of chemical inducer (Fig. 5 [B]). Measurements after 90 min suggest that although the linearity is somewhat reduced (R^2^ = 0.9016), reporter fluorescence is not yet saturated at concentrations of DTT up to 2 mM.

**Fig. 6:**
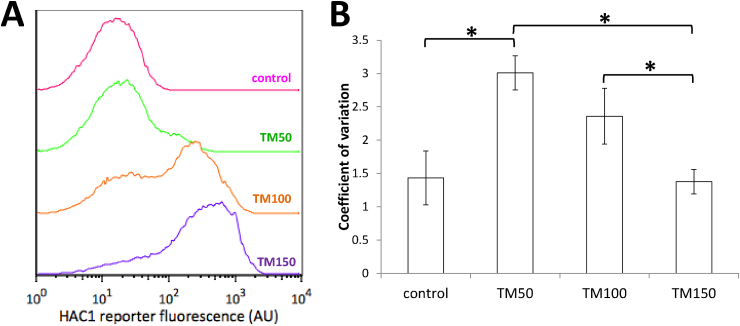
**UPR activation in the yeast cell population with increasing concentrations of tunicamycin**. [A] HAC1 reporter fluorescence during in the absence of tunicamycin (DMSO, pink), and at 50 (TM50, green), 100 (TM100, orange), and 150 (TM150, blue) ng/mL. Wild-type yeast cells were measured with flow cytometry 2.5 h after treatment. [B] The coefficient of variation (standard deviation/mean) of the results in [A]. The error bars represent the standard deviation of the coefficient of variation of three individual replicates (n = 3). Student's t-tests were carried out to determine significant differences between conditions; asterisk indicate a p-value < .05.

We then used the reporter to measure UPR activation across the yeast cell population at different concentrations of tunicamycin to characterise the behaviour at different levels of ER stress (Fig. 6 [A]). Concentrations of tunicamycin as low as 50 ng/mL (TM50) could activate UPR in a subset of the population. Interestingly, cells displayed a much wider range of fluorescence values at TM50 and TM100, than at TM150. We quantified this by calculating the coefficient of variation; results showed a statistically significant increase in the coefficient of variation between the control and TM50 and a near significant increase between the control and TM100 (p = .0509). The coefficient of variation was significantly reduced at TM150, compared to both of the lower concentrations (Fig. 6 [B]).

**Fig. 7:**
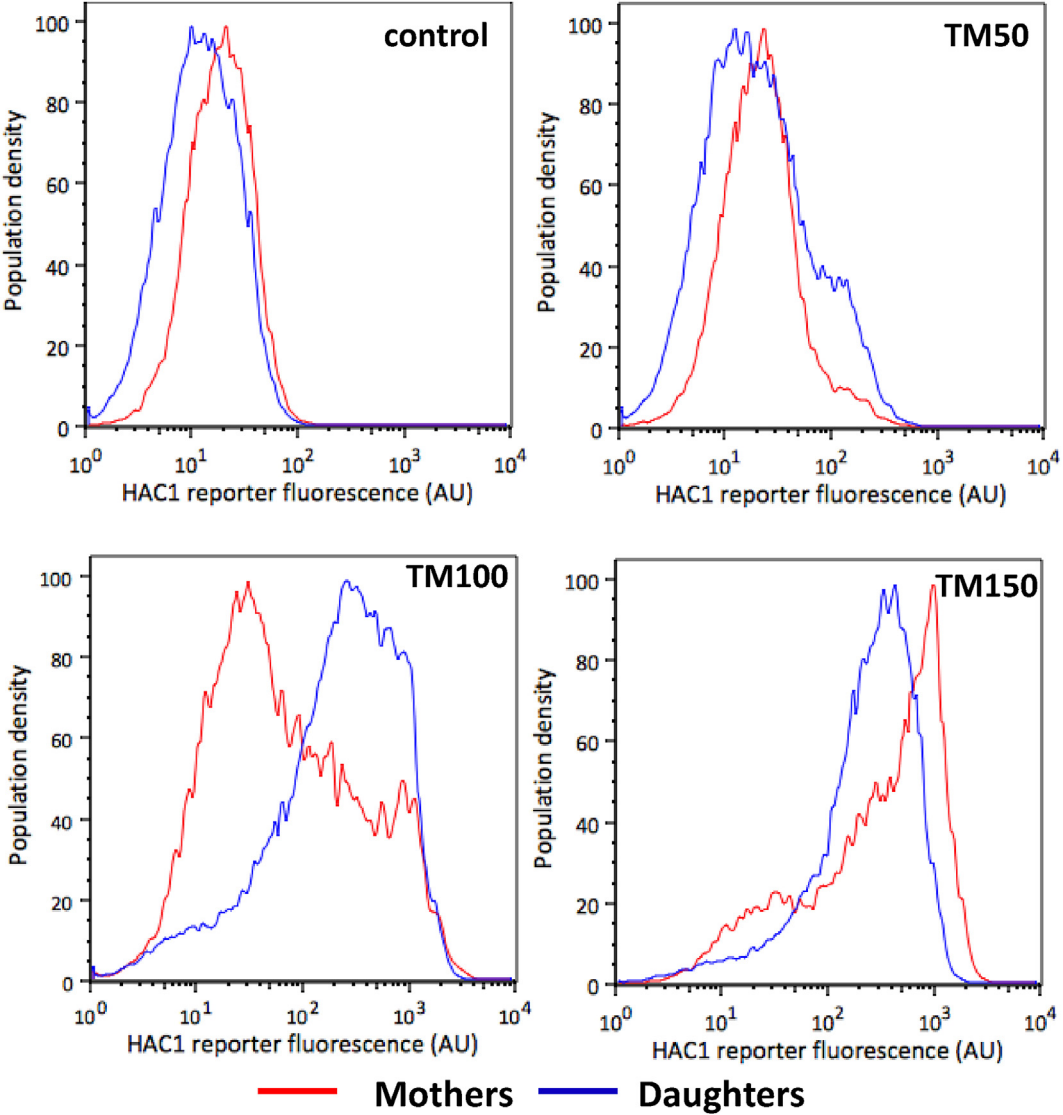
**UPR activation in mother and daughter cell populations.** ConA staining was used to distinguish mother (red) and daughter (blue) subpopulations treated with different concentrations of tunicamycin. The UPR activation was measured by the *HAC1* fluorescence reporter.

Considering our previous observation that the ER is asymmetrically divided during low (TM100) ER stress through the ERSU pathway (Fig. 3), we wondered if the population heterogeneity in UPR activation under these conditions was also attributable to mother and daughter subpopulations, therefore connecting the asymmetric division of the ER with UPR activity. We used ConA staining to distinguish mother and daughter cell subpopulations and compared reporter expression between the two groups (Fig. 7). Results showed that the reporter expression was strongly activated in daughter cells, when compared to mother cells, at low (TM100) ER stress levels. At higher (TM150) ER stress levels, however, both mother and daughter subpopulations uniformly activated expression of the UPR reporter. As the ERSU pathway is active in mother cells at low stress levels (Section 4.2), it suggests that asymmetric division is sufficient for them to circumvent UPR activation, but that both the ERSU and the UPR are required at medium (TM150) stress levels. However, the shift in reporter fluorescence from DMSO to TM100 treatment in daughter cells also implies that inheriting less ER necessitates activation of the UPR at lower ER stress levels.

**Fig. 8:**
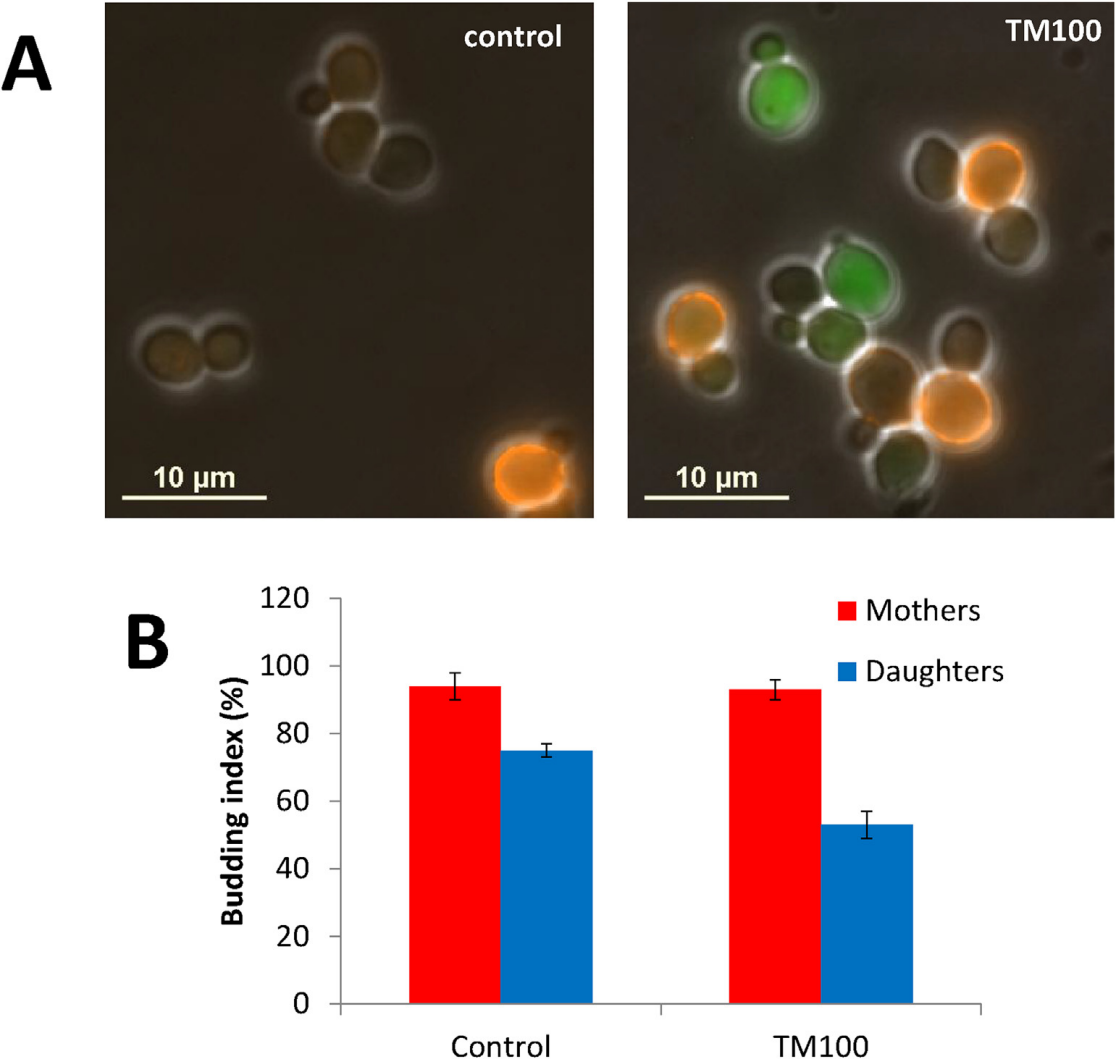
**The effect of low stress on the budding index of mother and daughter cells.** [A] Cells, grown in YPD for 3 h with either no (DMSO) or low (TM100) stress, were treated with ConA staining and assessed for budding with fluorescence microscopy. [B] The budding index of mother (red bars) and daughter (blue bars) subpopulations was calculated using a minimum of 100 cells. Error bars indicate standard deviation (n = 3).

As homeostatic pathways, such as the UPR, are activated to mediate adaptation, we sought to examine the effects on growth rate of differential UPR activation between mother and daughter cell subpopulations during low ER stress. One method to do so is to quantify the budding index – at the beginning of division, yeast cells develop a bud at the cell surface, which gives rise to the daughter cell at the end of the cell cycle. The budding index is the proportion of budded cells to total cells in an exponentially growing culture and, as a measure of progression through the cell cycle, signifies growth [43]. Using ConA staining to differentiate mothers from daughters, we measured the budding index by microscopy (Fig. 8). Under normal conditions (DMSO), daughters have a lower budding index than mother cells (mother cells 94 ± 4%, daughters 75 ± 2%), a phenomenon known as daughter delay [44]. Crucially, under low ER stress (TM100), the budding index of daughter cells is decreased further (mothers 93 ± 3%; daughters 53 ± 4%) while mothers are not significantly affected. Therefore, asymmetric division under low stress leads to lower UPR activation and unaltered cell division in mother cells, but increases UPR activation and lowers budding in daughter cells.

### 4.4 Mathematical modelling of the population effects of asymmetric division suggests that asymmetric division allows short-term population benefit before the long-term adaptation from the UPR

Our experimental results have revealed that, under low stress, asymmetric division leads to lower UPR activation and unaltered cell division in mother cells, and also to increased UPR activation and reduced budding rates in daughter cells. The net-effect of a daughter cell division is an additional mother cell, while the net-effect of a mother cell division is an additional daughter cell. Due to this relationship, the growth of one subpopulation depends on the division rate of the other subpopulation, and conditions that mainly affect the division rate of one subpopulation, e.g. low ER stress delaying daughter cell division, might have an unexpected impact on the growth of both subpopulations. We therefore developed a mathematical model that accounts for this interaction (Section 3.1), which can be used to calculate the impact of such conditions to help understand the population-wide changes during the adaptation to low ER stress.

The model, when simulated under the physiologically relevant condition ${\text{k}}_{\text{M}}{\text{k}}_{\text{D}}\geq {\text{d}}_{\text{M}}{\text{d}}_{\text{D}}$ , shows that the daughter-to-mother cell ratio converges to a constant value, called hereafter the steady-state ratio. (Fig. 2).

**Fig. 9:**
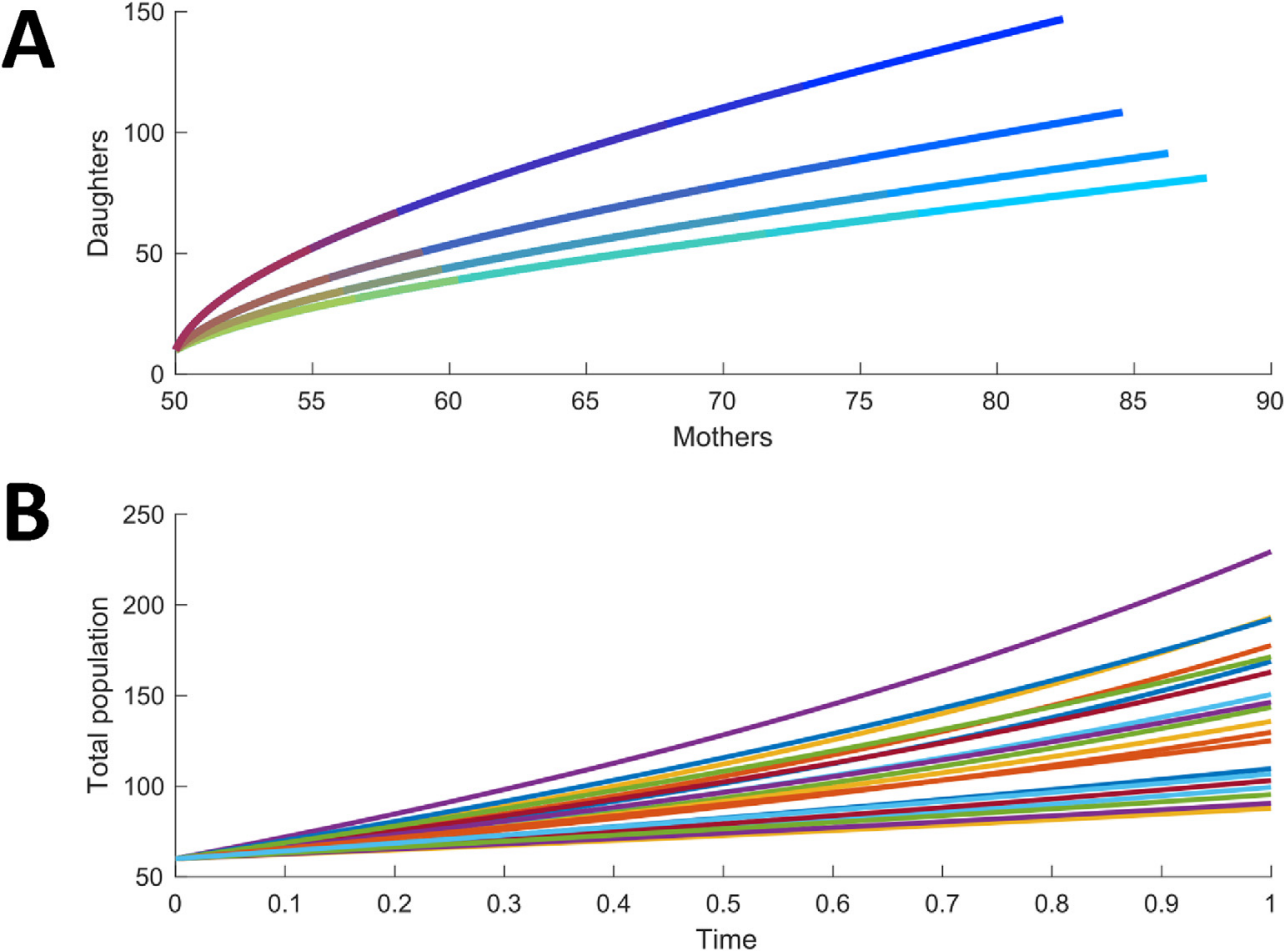
**The effect of ER stress and asymmetric inheritance on the mother-daughter populations.** [A] Simulated number of daughters and mothers in the population for different values of $k_{P}$ (ER stress) and a (ER asymmetric inheritance). Lines with more red indicate higher degrees of ER stress, lines with more green indicate less ER inheritance asymmetry. [B] Time evolution of total number of cells for populations with different combinations of $k_{P}$ (ER stress) and a (ER asymmetric inheritance).

To examine the effects of stress on the daughter-to-mother ratio and the total population growth rate, we considered increasing values for the concentration of tunicamycin in the presence of different degrees of asymmetric inheritance (see Appendix B for Matlab file). Experimental measurements of population growth rates (section 4.1, Fig. 3) were used to estimate $k_{P}$ at different concentrations of tunicamyin. To reflect ER asymmetric inheritance, we explored several values of the parameter a. The maximum value of a was calculated from the budding index data (section 4.3, Fig. 8) as the ratio of the budding index in daughters divided by that of mothers; this was then multiplied by various fractions in order to simulate increasing degrees of ER inheritance asymmetry. Based on this, the model was simulated for various combinations of $k_{P}$ and a. Our simulation results indicate that increasing ER inheritance asymmetry leads to an accumulation of daughter cells in the population (Fig. 9 [A]), as expected given the delay in cell cycle induced by inheritance of a lower amount of ER. At any degree of asymmetry, however, the population trajectory does not change in response to tunicamycin (i.e. ratio of daughter to mother cells stays the same), but the population grows more slowly with higher ER stress, meaning that the total population number during the simulation is lower. Fig. 9 [B] shows the total population size at various combinations of ER stress and asymmetry.

We then went on to calculate the growth rates of the mother and daughter subpopulations in the absence of stress and at 100 ng/mL tunicamycin using the data from sections 4.1, 4.3 and Equations 7 and 8 (Table 1). The ratio of budding indices for daughter and mother cells (section 4.3, Fig. 8) were used to calculate a. The values obtained (0.80 and 0.57 for no stress and 100 ng/mL tunicamycin, respectively), reflect the relatively larger decrease in growth rate of daughter cells versus mother cells and confirms the existence of ER inheritance asymmetry at low ER stress. The population growth rate data (section 4.1, Fig. 3) and the calculated values for a were then used to calculate $k_{M}$ and $k_{D}$. Consistent with our experimental observations, the values of $k_{M}$ do not substantially differ between the conditions, while the value of $k_{D}$ is substantially reduced under low ER stress.

**Table 1:** Calculated growth rates for mother and daughter subpopulations.

	No stress	100 ng/mL tunicamycin
Measured $k_{p}$	1	0.84
Calculated a	0.80	0.57
Calculated $k_{D}$	0.89	0.63
Calculated $k_{M}$	1.12	1.11

## 5 Discussion and conclusions

Homeostasis and its maintenance in changing environments are key features of all living organisms. The importance of adaptation to life has resulted in the evolution of a range of signalling pathways to monitor the environment and adjust the physiology of the cell in response. Not all such pathways activate transcription; increasingly, there are a variety of other mechanisms that are being discovered. In the case of the ER, one non-transcriptional mechanism is the ERSU pathway, which causes asymmetric partitioning of the ER during cell division under stress. We sought to better understand the interplay between ERSU and the canonical UPR signalling pathway that modifies transcription during adaptation. In particular, we focused on conditions of low ER stress, which are more physiologically relevant than experimental protocols that use high concentrations of chemical stressors to study the molecular basis of signalling. Understanding how responses are altered with the degree of stress applied is important as environmental changes are rarely binary.

It was previously reported [23], [24] that the presence of high ER stress in yeast causes mother cells to retain damaged ER and prolong cytokinesis to allow population adaptation. In this case, if ERSU activity is eradicated by knocking out the essential Slt2 MAP kinase, both mother and daughter cells die, suggesting that it is an essential pathway for adaptation. Similarly, UPR-deficient cells also do not survive the activation of strong ER stress (Fig. 3). In this study, we have shown that the same UPR and ERSU mechanisms operate under low stress as have been observed in high stress conditions. This observation confirms that both pathways are important for adaptation under physiologically relevant environmental changes. Further, we showed that the interaction of ERSU and UPR has interesting consequences for the adaptation of different sub-populations. Under low stress, activation of the ERSU pathway leads to the retention of ER by mother cells, causing a decrease in ER content in daughter cells. The asymmetric inheritance necessitates UPR activation and a delayed cell cycle in daughter cells so that they can recover functionality before commencing growth.

The existence of two potentially redundant pathways and the difference in sub-population responses led us to further explore the consequences of ERSU and UPR for the population composition. Therefore, we developed a mathematical model to understand how the two signalling pathways affect mother and daughter cells and used our experimental data to choose parameters for simulation. The results suggest that asymmetric inheritance of the ER affects the ratio of daughter-to-mother cells in the population, but that this converges to a stable ratio over time, suggesting the impact of ERSU is primarily on the population composition, with very little long-term effect on growth rate. Interestingly, ER stress level only affects the population growth rate, i.e. populations in different stress conditions grow along the same trajectory and reach the same ratio of daughter-to-mother cells, but take longer to accumulate as ER stress increases. Therefore, activation of the ERSU pathway may be a rapid mechanism, to enable the population to continue growing in the short-term, while UPR signalling occurs to facilitate a return to homeostasis over longer time scales.

The results of this study demonstrate the importance of understanding pathway interplay in adaptation to environmental changes and illustrate the importance of single-cell measurements for identifying underlying differences in the response of sub-populations. Moreover, yeast, as a simple eukaryote, is an ideal platform for such explorations as it uses many of the same signalling pathways as higher organisms, but is simpler to genetically modify and easier to manipulate experimentally. Even though the UPR has more branches in higher eukaryotes, we speculate that the interplay between UPR and ERSU has similar consequences in mammalian cells as in yeast. Given the role of UPR activation in diseases, such as neurodegeneration and cancer, examining this interplay could lead to a better understanding of disease progression and suggest new intervention points for treatment. In addition, asymmetric division of other cellular resources (mitochondria, protein content, etc.) in different environments may play a role in adaptation alongside canonical signalling pathways for other types of stress. This area has only begun to be explored and may lead to new insights into how multiple adaptation strategies can be integrated to enable population survival under challenging conditions.

## Conflicts of interest

The authors have no conflict of interest to declare.

## Figures and Tables

**Table A1:** Yeast Strains

Name	Genotype	Source
BY4741	MATa; his3Δ1, leu2Δ0, ura3Δ0, met15Δ0	GE Dharmacon
Δhac1	BY4741 hac1Δ::kanMX4	GE Dharmacon
FJ4741.1	BY4741 his3Δ::pFJ1	This work
FJ4741.17	BY4741 his3Δ::pFJ17	This work

**Table A2:** Plasmids used in this study

Name	Details	Reference
pRS403	His3 integration plasmid	Sikorski et al., 1989 [4]
pPM28	ero-GFP template	Merksamer et al., 2008 [2]
pPM47	UPRE template	Merksamer et al., 2008 [2]
pMaM175	sfGFP template	Khmelinskii et al., 2011 [3]
pFJ1	pRS403 with UPRE-Ubi-E-sfGFP	This work
pFJ17	pRS403 with eroGFP	This work

**Fig. A1:**
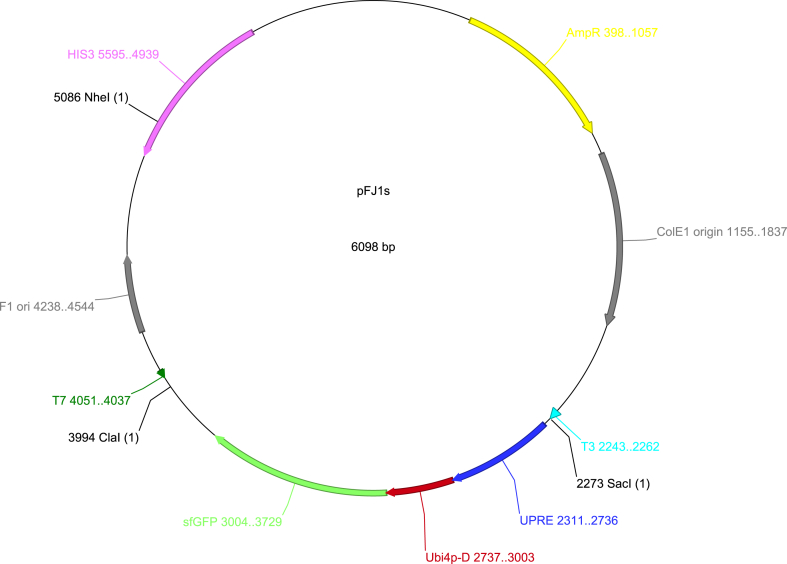
**Genomic integration plasmid for the novel, dynamic, transcriptional UPR reporter.** The UPRE promoter, ubiquitin domain, Superfolder GFP and the CYC1 terminator were fused by overlap extension PCR and cloned into the multiple cloning site of pRS403 using *Sac*I and *Cla*I (Sikorski et al., 1989, Merksamer et al., 2008, Khmelinskii et al., 2011). *Nhe*I was used for linearization prior to yeast transformation.

## Appendices

### Appendix A Supplementary Experimental Methods (yeast strains, plasmids, and the construction of the UPR reporter)

Construction of the dynamic UPR reporter:

Briefly, the UPR reporter is based on a previous transcriptional UPR reporter [1] and consists of the 4X UPR response element (UPRE) from pPM47 [2] and a ubiquitin domain fused to the gene for a yeast codon-optimised green fluorescent protein (GFP) [3]. The ubiquitin domain enables rapid turnover of the GFP, allowing the monitoring of rapid changes in gene expression.

References

1. Cox JS, Walter P: A novel mechanism for regulating activity of a transcription factor that controls the unfolded protein response. *Cell* 1996, 87 (3):391–404.
2. Merksamer PI, Trusina A, Papa FR: Real-time redox measurements during endoplasmic reticulum stress reveal interlinked protein folding functions. *Cell* 2008, 135 (5):933–947.
3. Khmelinskii A, Meurer M, Duishoev N, Delhomme N, Knop M: **Seamless gene tagging by endonuclease-driven homologous recombination.** *Plos One* 2011, **6** (8).
4. Sikorski RS, Hieter P: A system of shuttle vectors and yeast host strains designed for efficient manipulation of DNA in *Saccharomyces cerevisiae Genetics* 1989, 122 (1):19–27.
5. Scott M, Klumpp S, Mateescu EM, Hwa T: **Emergence of robust growth laws from optimal regulation of ribosome synthesis.** *Molecular systems biology* 2014, **10** (8).

### Appendix B Supplementary data

Matlab code for simulations.

mmc1

mmc2
